# Supplementary material for: Repeated sleep disruption in mice leads to persistent shifts in the fecal microbiome and metabolome
Source: PLoS One. 2020 Feb 20;15(2):e0229001. doi: 10.1371/journal.pone.0229001 (PMC7032712; doi:10.1371/journal.pone.0229001)
Supplement: S1 Fig — A,B) There was a significant increase in the 24-hour totals of total sleep bouts (A) and non-rapid eye movement sleep (NREM) bouts (B) during the sleep disruption protocol in the sleep disruption group. C) Rapid eye movement (REM) bouts were decreased during the sleep disruption protocol, and increased on the first day of recovery in the sleep-disrupted group. D,E,F) Median sleep bout duration (D) and NREM bout duration (E) were significantly decreased in the sleep-disrupted group during the protocol, while the median REM bout duration (F) was unaffected in all days except for on S5. G) There was no change in 24-hour NREM delta power due to sleep disruption. Abbreviations: BL, baseline; S, sleep disruption; R, recovery; ZT, zeitgeber time. n = 3-4/group. *p < 0.05, **p < 0.01, ***p < 0.001 (Bonferroni post hoc test); +p < 0.05 (overall effect of sleep disruption, Mixed-effects model); •p < 0.05 (overall effect of Time, Mixed-effects model); #p < 0.05 (Sleep DisruptionxTime interaction, Mixed-effects model). (PDF) [file pone.0229001.s001.pdf]

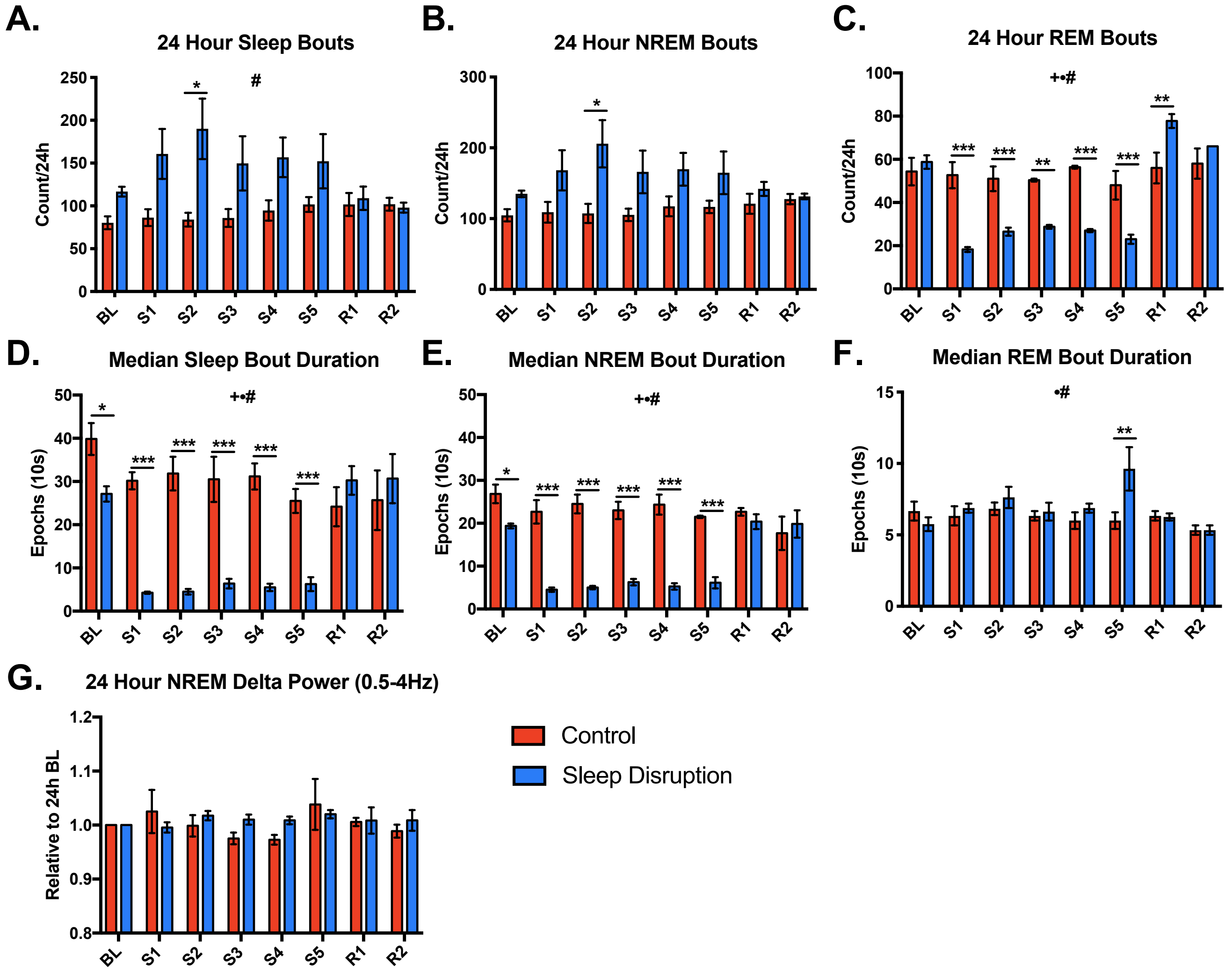

A.
24 Hour Sleep Bouts
Count/24h
B.
24 Hour NREM Bouts
Count/24h
C.
24 Hour REM Bouts
Count/24h
+•#
D.
Median Sleep Bout Duration
Epochs (10s)
+•#
E.
Median NREM Bout Duration
Epochs (10s)
+•#
F.
Median REM Bout Duration
Epochs (10s)
•#
G.
24 Hour NREM Delta Power (0.5-4Hz)
Relative to 24h BL
BL S1 S2 S3 S4 S5 R1 R2
Control
Sleep Disruption
