# Supplementary figures and images for: Repeated sleep disruption in mice leads to persistent shifts in the fecal microbiome and metabolome
Source: PLoS One. 2020 Feb 20;15(2):e0229001. doi: 10.1371/journal.pone.0229001 (PMC7032712; doi:10.1371/journal.pone.0229001)

**A.**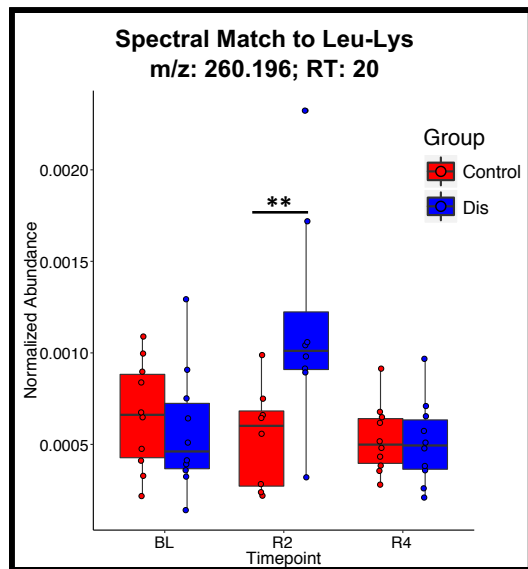**C.**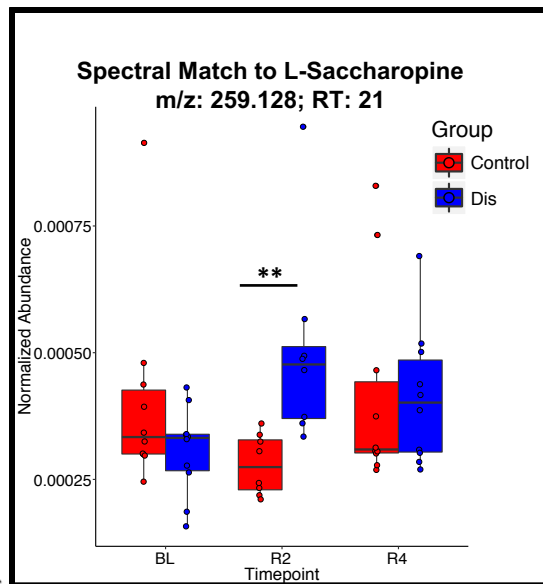**B.**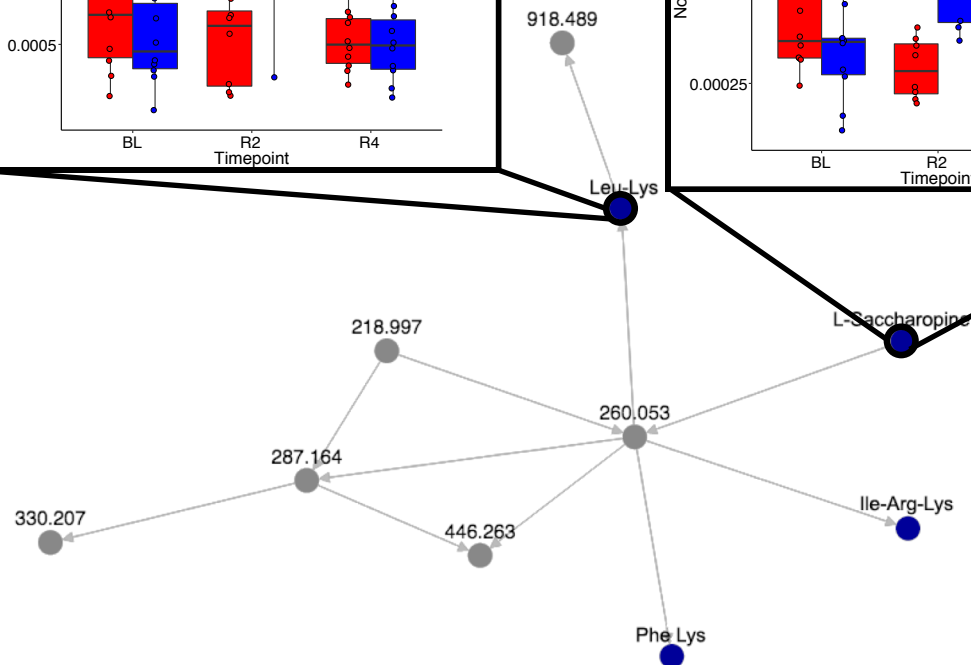

Supplement: S2 Fig — A) Normalized abundance (peak intensity normalized to total ion count) of a metabolite with a spectral match to Leu-Lys was increased at day 2 post-sleep disruption. B) Global Natural Products Social Molecular Networking (GNPS)-generated molecular network containing multiple annotated (blue) and unannotated (grey) clusters. Numbers next to grey clusters indicate average parent mass of the spectra in the cluster. Metabolites that were above the threshold variable importance in Variable Selection Using Random Forests (VSURF) analysis are outlined with a black circle. Length of grey lines connecting clusters indicates relative similarity of the MS2 spectra. Arrowheads point towards clusters with a larger m/z. C) Normalized abundance of a metabolite with a spectral match to L-saccharopine was increased at day 2 post-sleep disruption (R2). Boxes indicate median, 25th and 75th quantiles; whiskers indicate 2*IQR from edges of box. Abbreviations: BL, baseline; R2, day 2 post-sleep disruption; R4, day 4 post-sleep disruption; Dis, Sleep Disruption; VI, variable importance; m/z, mass to charge ratio; RT, retention time (seconds). n = 8-10/group. *p < 0.05, **p < 0.01 (Wilcoxon-Rank Sum test). (PDF) [file pone.0229001.s002.pdf]

**A.**

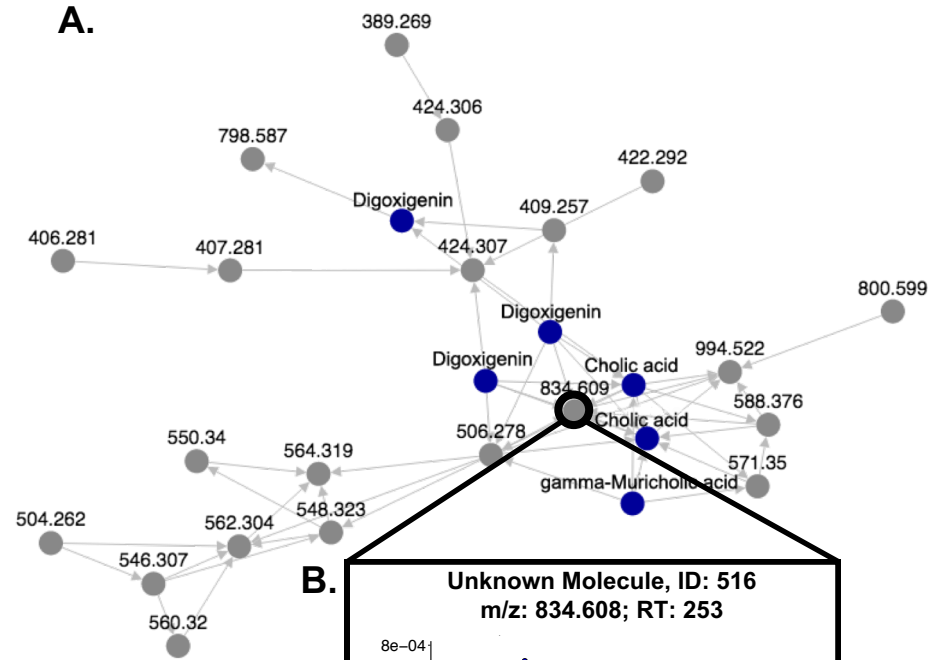

**B.**

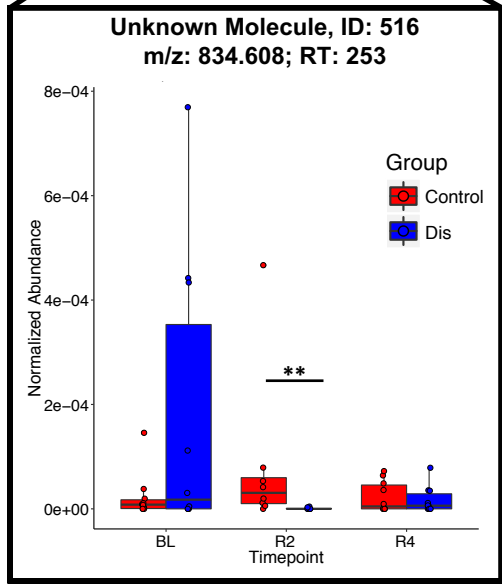

**C.**

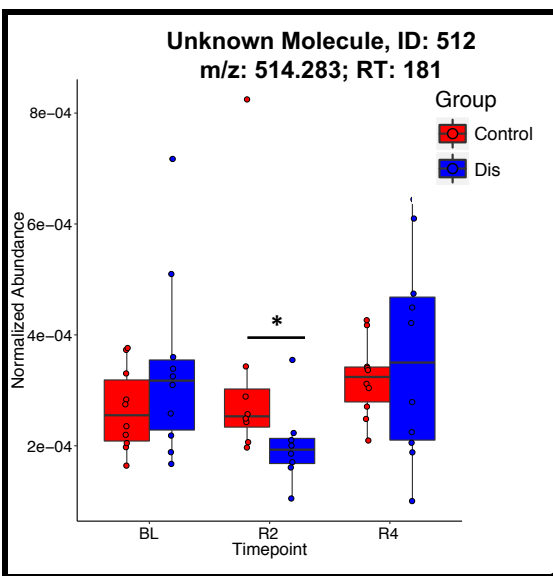

**D.**

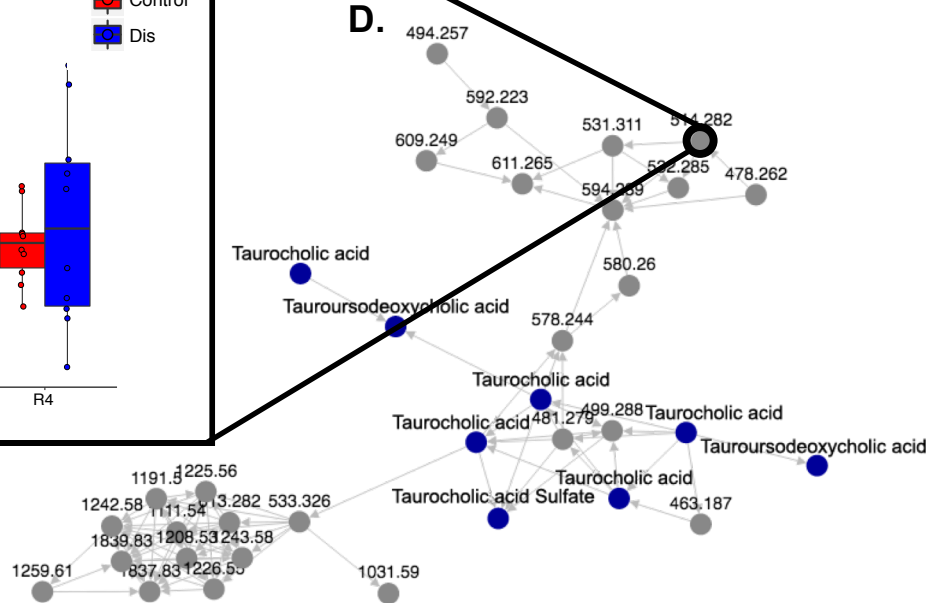

Supplement: S3 Fig — A,D) Global Natural Products Social Molecular Networking (GNPS)-generated molecular networks containing multiple annotated (blue) and unannotated (grey) clusters. Numbers next to grey clusters indicate average parent mass of the spectra in the cluster. Metabolites that were above the threshold variable importance in Variable Selection Using Random Forests (VSURF) analysis are outlined with a black circle. Length of grey lines connecting clusters indicates relative similarity of the MS2 spectra. Arrowheads point towards clusters with a larger m/z. B) Normalized abundance (peak intensity normalized to total ion count) of an unannotated metabolite with ID 516 was decreased at day 2 post-sleep disruption (R2). C) Normalized abundance (peak intensity normalized to total ion count) of an unannotated metabolite with ID 512 was decreased at R2. Boxes indicate median, 25th and 75th quantiles; whiskers indicate 2*IQR from edges of box. Abbreviations: BL, baseline; R2, day 2 post-sleep disruption; R4, day 4 post-sleep disruption; Dis, Sleep Disruption; VI, variable importance; m/z, mass to charge ratio; RT, retention time (seconds). n = 8-10/group. *p < 0.05, **p < 0.01 (Wilcoxon-Rank Sum test). (PDF) [file pone.0229001.s003.pdf]

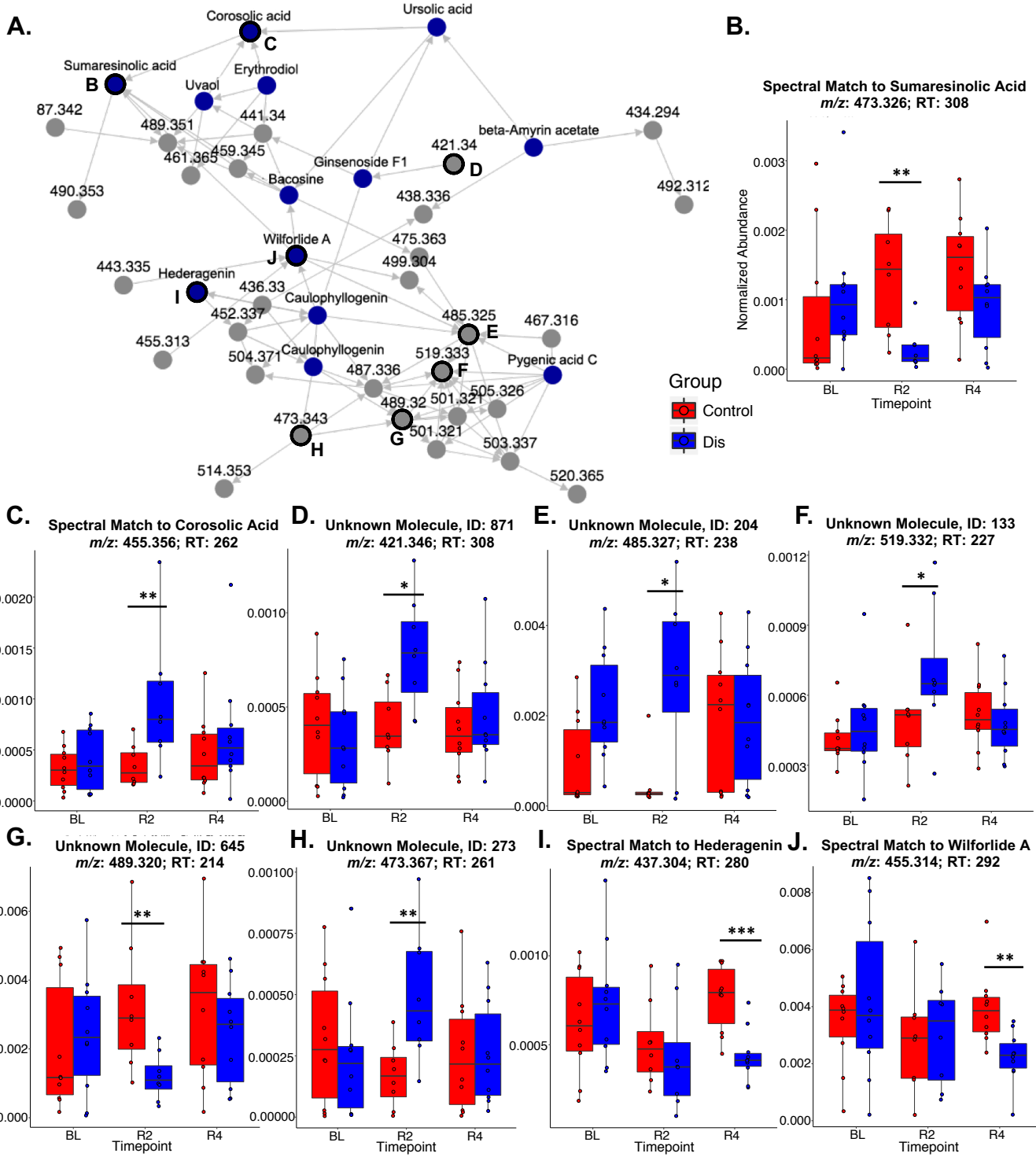

Supplement: S4 Fig — A) Global Natural Products Social Molecular Networking (GNPS)-generated molecular network containing multiple annotated (blue) and unannotated (grey) clusters. Numbers next to grey clusters indicate average parent mass of the spectra in the cluster. Metabolites that were above the threshold variable importance in Variable Selection Using Random Forests (VSURF) analysis are outlined with a black circle. Letters next to the black circles indicate the panel of the figure corresponding to the cluster. Length of grey lines connecting clusters indicates relative similarity of the MS2 spectra. Arrowheads point towards clusters with a larger m/z. B,C) Normalized abundance (peak intensity normalized to total ion count) of a metabolite with a spectral match to sumaresinolic acid (B) was decreased at day 2 post-sleep disruption, while a spectral match to corosolic acid (C) was increased at R2. D,E,F,H) Unannotated molecules with ID 871 (D), 204 (E), 133 (F), and 273 (H) were increased at R2. G,I,J) Unannotated molecule ID 645 (G) was decreased at R2. Metabolites matching hederagenin (I), and wilforlide A (J) were decreased at R4. Boxes indicate median, 25th and 75th quantiles; whiskers indicate points within 2*IQR from edges of box. Abbreviations: BL, baseline; R2, day 2 post-sleep disruption; R4, day 4 post-sleep disruption; Dis, Sleep Disruption; VI, variable importance; m/z, mass to charge ratio; RT, retention time (seconds). n = 8-10/group. *p < 0.05, **p < 0.01, ***p < 0.001 (Wilcoxon-Rank Sum test). (PDF) [file pone.0229001.s004.pdf]

**A.**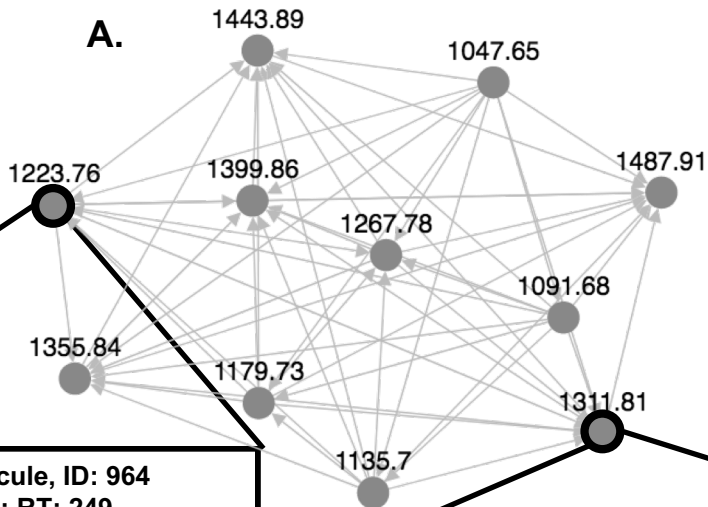**B.**

**Unknown Molecule, ID: 964**  
**m/z: 612.383; RT: 249**

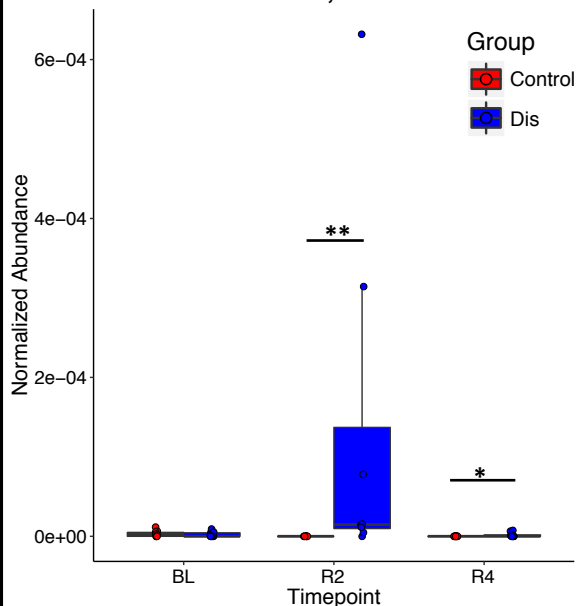**C.**

**Unknown Molecule, ID: 965**  
**m/z: 656.409; RT: 250**

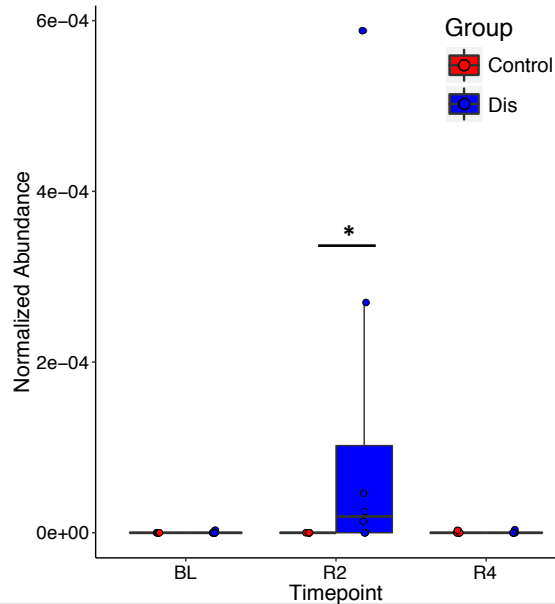

Supplement: S5 Fig — A) Global Natural Products Social Molecular Networking (GNPS)-generated molecular network containing multiple unannotated (grey) clusters. Numbers next to grey clusters indicate average parent mass of the spectra in the cluster. Metabolites that were above the threshold variable importance in Variable Selection Using Random Forests (VSURF) analysis are outlined with a black circle. Length of grey lines connecting clusters indicates relative similarity of the MS2 spectra. Arrowheads point towards clusters with a larger m/z. B) Normalized abundance (peak intensity normalized to total ion count) of an unannotated metabolite with ID 964 was increased at day 2 post-sleep disruption and at day 4 post-sleep disruption. C) Normalized abundance (peak intensity normalized to total ion count) of an unannotated metabolite with ID 965 was increased at day 2 post-sleep disruption but not day 4 post-sleep disruption. Boxes indicate median, 25th and 75th quantiles; whiskers indicate points within 2*IQR from edges of box. Abbreviations: BL, baseline; R2, day 2 post-sleep disruption; R4, day 4 post-sleep disruption; Dis, Sleep Disruption; VI, variable importance; m/z, mass to charge ratio; RT, retention time (seconds). n = 8-10/group. *p < 0.05, **p < 0.01 (Wilcoxon-Rank Sum test). (PDF) [file pone.0229001.s005.pdf]
