# Supplementary material for: Repeated sleep disruption in mice leads to persistent shifts in the fecal microbiome and metabolome
Source: PLoS One. 2020 Feb 20;15(2):e0229001. doi: 10.1371/journal.pone.0229001 (PMC7032712; doi:10.1371/journal.pone.0229001)
Supplement: S6 Fig — A) Heatmap of the 64 metabolites that were above threshold variable importance in Variable Selection Using Random Forests (VSURF) analysis. B) Variable importance (VI) scores of the top 25 suprathreshold metabolites (m/z_RT). Bold indicates metabolites that were annotated using Global Natural Products Social Molecular Networking (GNPS). C) VI scores of annotated metabolites. D) Normalized abundance (peak intensity normalized to total ion count) of an unannotated metabolite with ID 241 was increased in sleep-disrupted relative to control mice at both day 2 post-sleep disruption (R2) and day 4 post-sleep disruption (R4). E) An unannotated metabolite with ID 661 was trending towards an increase in sleep-disrupted compared to control at R2 and was increased at R4. F) An unannotated metabolite with ID 155 was trending towards a decrease in sleep-disrupted mice compared to control mice at R2 and was decreased at R4. Boxes indicate median, 25th and 75th quantiles; whiskers indicate 2*IQR from edges of box. Abbreviations: BL, baseline; R2, day 2 post-sleep disruption; R4, day 4 post-sleep disruption; VI, variable importance; Dis, sleep disruption; m/z, mass to charge ratio; RT, retention time (seconds). n = 8-10/group. *p < 0.05, **p < 0.01 (Wilcoxon-Rank Sum test). (PDF) [file pone.0229001.s006.pdf]

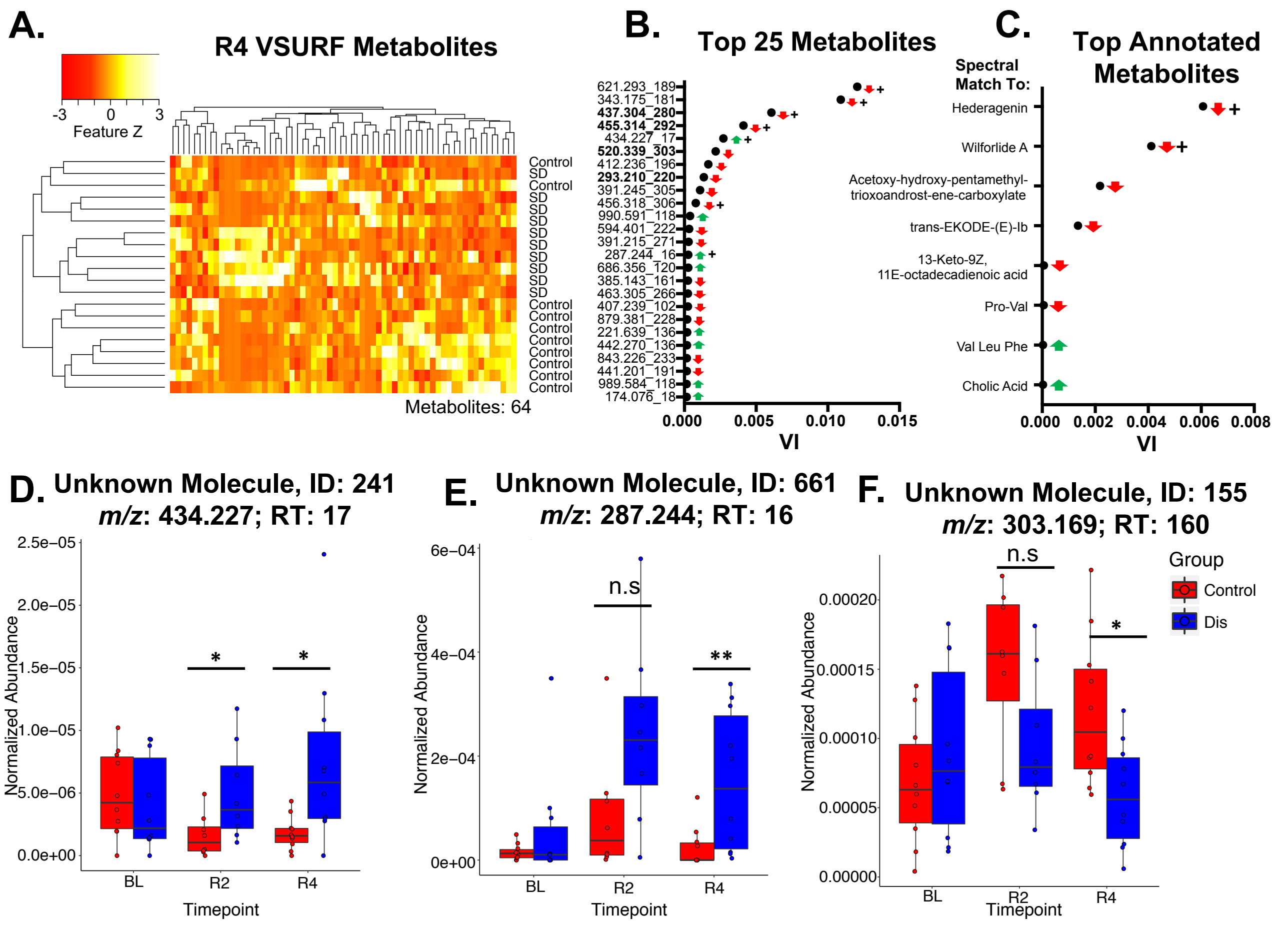
A.
R4 VSURF Metabolites
-3 0 3
Feature Z
Control
SD
Control
SD
SD
SD
SD
SD
SD
SD
SD
SD
Control
Control
Control
Control
Control
Control
Control
Control
Metabolites: 64
B.
Top 25 Metabolites
621.293_189
343.175_181
437.304_280
455.314_292
434.227_17
520.339_303
412.236_196
293.210_220
391.245_305
456.318_306
990.591_118
594.401_222
391.215_271
287.244_16
686.356_120
385.143_161
463.305_266
407.239_102
879.381_228
221.639_136
442.270_136
843.226_233
441.201_191
989.584_118
174.076_18
0.000 0.005 0.010 0.015
VI
C.
Top Annotated Metabolites
Spectral Match To:
Hederagenin
Wilforlide A
Acetoxy-hydroxy-pentamethyl-trioxoandrost-ene-carboxylate
trans-EKODE-(E)-Ib
13-Keto-9Z, 11E-octadecadienoic acid
Pro-Val
Val Leu Phe
Cholic Acid
0.000 0.002 0.004 0.006 0.008
VI
D.
Unknown Molecule, ID: 241
m/z: 434.227; RT: 17
Normalized Abundance
2.5e−05 2.0e−05 1.5e−05 1.0e−05 5.0e−06 0.0e+00
BL R2 R4
Timepoint
E.
Unknown Molecule, ID: 661
m/z: 287.244; RT: 16
n.s
Normalized Abundance
6e−04 4e−04 2e−04 0e+00
BL R2 R4
Timepoint
F.
Unknown Molecule, ID: 155
m/z: 303.169; RT: 160
n.s
Normalized Abundance
0.00020 0.00015 0.00010 0.00005 0.00000
BL R2 R4
Timepoint
Group
Control
Dis
