# Supplementary material for: Repeated sleep disruption in mice leads to persistent shifts in the fecal microbiome and metabolome
Source: PLoS One. 2020 Feb 20;15(2):e0229001. doi: 10.1371/journal.pone.0229001 (PMC7032712; doi:10.1371/journal.pone.0229001)
Supplement: S2 Table — DESeq2 was performed at each taxonomic level to determine taxa differentially abundant between sleep disrupted and control groups. Taxa significant at an FDR < 0.1 are listed below as the mean relative abundance ± SEM, along with Benjamini Hochberg-adjusted p values. Fold difference: (Sleep Disruption-Control)/Control. Abbreviations: Dis, Sleep Disruption; Con, Control. n = 8-10/group. (DOCX) [file pone.0229001.s008.docx]

**Table S2. Differentially Abundant Bacterial Taxa Post-Sleep Disruption**

**Day 2 Post-Sleep Disruption**

| Taxon | Control  Relative Abundance | Sleep Disrupted  Relative Abundance | Fold Difference  (Dis vs Con) | Adjusted p |
| --- | --- | --- | --- | --- |
| Phylum Actinobacteria | 0.0050 + 0.0020 | 0.0006 + 0.0002 | -0.8800 | 0.0004 |
| Class Actinobacteria | 0.0044 + 0.0020 | 0.0001 + 0.0001 | -0.9773 | 0.012 |
| Order Bifidobacteriales | 0.0042 + 0.0021 | 0.0001 + 0.0001 | -0.9762 | 0.028 |
| Family Bifidobacteriaceae | 0.0042 + 0.0021 | 0.0001 + 0.0001 | -0.9762 | 0.032 |
| Genus Bifidobacterium | 0.0042 + 0.0021 | 0.0001 + 0.0001 | -0.9762 | 0.046 |
| Phylum Firmicutes | 0.370 + 0.036 | 0.558 + 0.072 | 0.508 | 0.002 |
| Class Bacilli | 0.027 + 0.010 | 0.001 + 4.2e-4 | -0.963 | 1.9e-6 |
| Order Lactobacillales | 0.013 + 0.008 | 0.001 + 4.2e-4 | -0.923 | 0.003 |
| Family Lactobacillaceae | 0.013 + 0.008 | 0.001 + 3.7e-4 | -0.923 | 0.023 |
| Genus Lactobacillus | 0.012 + 0.008 | 0.001 + 3.7e-4 | -0.917 | 0.047 |
| Order Turicibacterales | 0.014 + 0.008 | 0.000 + 0.000 | -1.000 | 0.015 |
| Family Turicibacteraceae | 0.014 + 0.008 | 0.000 + 0.000 | -1.000 | 0.003 |
| Genus Turicibacter | 0.014 + 0.008 | 0.000 + 0.000 | -1.000 | 0.047 |
| Class Clostridia | 0.340 + 0.037 | 0.553 + 0.072 | 0.627 | 0.051 |
| Order Clostridiales | 0.340 + 0.037 | 0.553 + 0.072 | 0.627 | 0.028 |
| Family Clostridiaceae, Genus unknown | 0.003 + 0.002 | 0.001 + 0.0005 | -0.667 | 0.047 |

**Day 4 Post-Sleep Disruption**

| Taxon | Control  Relative Abundance | Sleep Disrupted Relative Abundance | Fold Difference  (Dis vs Con) | Adjusted p |
| --- | --- | --- | --- | --- |
| Phylum Actinobacteria | 0.0038 + 0.0014 | 0.0006 + 0.0002 | -0.8421 | 0.004 |
| Class Actinobacteria | 0.0029 + 0.0014 | 4.9e-5 + 3.8e-5 | -0.9831 | 0.005 |
| Order Bifidobacteriales | 0.0028 + 0.0013 | 4.9e-5 + 3.8e-5 | -0.9825 | 0.002 |
| Family Bifidobacteriaceae | 0.0028 + 0.0013 | 4.9e-5 + 3.8e-5 | -0.9825 | 0.015 |
| Genus Bifidobacterium | 0.0028 + 0.0013 | 4.9e-5 + 3.8e-5 | -0.9825 | 0.005 |
| Phylum Firmicutes | 0.439 + 0.015 | 0.475 + 0.073 | 0.082 | 0.045 |
| Order Turicibacterales | 0.010 + 0.007 | 0.000 + 0.000 | -1.00 | 0.087 |

**Table S2. Differentially Abundant Bacterial Taxa Post-Sleep Disruption.** DESeq2 was performed at each taxonomic level to determine taxa differentially abundant between sleep disrupted and control groups. Taxa significant at an FDR < 0.1 are listed below as the mean relative abundance + SEM, along with Benjamini Hochberg-adjusted *p* values. Fold difference: (Sleep Disruption-Control)/Control. Abbreviations: Dis, Sleep Disruption; Con, Control. *n* = 8-10/group.
