# Supplementary material for: Repeated sleep disruption in mice leads to persistent shifts in the fecal microbiome and metabolome
Source: PLoS One. 2020 Feb 20;15(2):e0229001. doi: 10.1371/journal.pone.0229001 (PMC7032712; doi:10.1371/journal.pone.0229001)
Supplement: S3 Table — Metabolites that were above the VSURF threshold variable importance (‘suprathreshold’) from each timepoint are listed above, along with the feature’s ID, m/z ratio, retention time, annotated name (if any), fold difference in the sleep-disrupted group compared to the control group, unadjusted Wilcoxon Rank-Sum p value, and Benjamini Hochberg (FDR)-adjusted p value. Bold indicates adjusted p values that are below the FDR of 0.1. Fold difference: (Sleep Disruption-Control)/Control. Abbreviations: Dis, sleep disruption; Con, control; MZ, mass to charge ratio; RT, retention time; N/A, not annotated; Inf, infinity. n = 8-10/group. (DOCX) [file pone.0229001.s009.docx]

**Table S3. VSURF Suprathreshold Metabolites**

**Day 2 Post-Sleep Disruption**

| VSURF Rank | Feature ID | M/Z | RT (sec) | Annotated Name | Fold Difference (Dis vs Con) | P Value | Adj. P Value |
| --- | --- | --- | --- | --- | --- | --- | --- |
| 1 | 964 | 612.383 | 249 | N/A | 9638.33 | 0.0025 | **0.0218** |
| 2 | 83 | 373.274 | 298 | N/A | 3.80 | 0.0030 | **0.0218** |
| 3 | 908 | 511.303 | 237 | N/A | -0.54 | 0.0011 | **0.0218** |
| 4 | 516 | 834.608 | 253 | N/A | -0.99 | 0.0037 | **0.0218** |
| 5 | 79 | 259.128 | 21 | L-Saccharopine | 0.80 | 0.0006 | **0.0218** |
| 6 | 998 | 619.344 | 271 | N/A | 4.32 | 0.0019 | **0.0218** |
| 7 | 780 | 159.077 | 17 | N/A | 2.69 | 0.0047 | **0.0218** |
| 8 | 809 | 177.958 | 17 | N/A | 6.24 | 0.0019 | **0.0218** |
| 9 | 13 | 591.317 | 194 | Urobilin | -0.38 | 0.0104 | **0.0262** |
| 10 | 727 | 513.356 | 260 | N/A | 2.97 | 0.0030 | **0.0218** |
| 11 | 351 | 994.521 | 223 | N/A | -0.63 | 0.0235 | **0.0391** |
| 12 | 1029 | 910.906 | 182 | N/A | -0.91 | 0.0159 | **0.0331** |
| 13 | 65 | 452.312 | 205 | N/A | 0.51 | 0.0047 | **0.0218** |
| 14 | 582 | 347.095 | 17 | Ala-Glu-Lys | 4.08 | 0.0207 | **0.0349** |
| 15 | 653 | 162.055 | 30 | N/A | -0.16 | 0.1049 | 0.1105 |
| 16 | 1075 | 427.321 | 260 | N/A | -0.43 | 0.0047 | **0.0218** |
| 17 | 467 | 330.263 | 152 | N/A | 0.58 | 0.0499 | **0.0596** |
| 18 | 939 | 330.264 | 304 | N/A | 0.62 | 0.0499 | **0.0596** |
| 19 | 41 | 593.333 | 269 | N/A | 17.86 | 0.0047 | **0.0218** |
| 20 | 1014 | 431.274 | 256 | N/A | -0.45 | 0.0047 | **0.0218** |
| 21 | 52 | 355.263 | 279 | N/A | 2.50 | 0.0207 | **0.0349** |
| 22 | 503 | 304.15 | 18 | N/A | 1.74 | 0.0070 | **0.0236** |
| 23 | 889 | 471.306 | 239 | N/A | -0.42 | 0.0104 | **0.0262** |
| 24 | 589 | 613.288 | 193 | N/A | -0.46 | 0.0104 | **0.0262** |
| 25 | 564 | 373.274 | 270 | Cholic Acid | -0.48 | 0.0070 | **0.0236** |
| 26 | 622 | 417.336 | 230 | N/A | 0.53 | 0.0104 | **0.0262** |
| 27 | 906 | 491.279 | 156 | N/A | -0.39 | 0.0379 | **0.0489** |
| 28 | 243 | 176.103 | 17 | N/A | 1.87 | 0.0047 | **0.0218** |
| 29 | 207 | 355.264 | 223 | Cholic Acid | 1.67 | 0.0650 | **0.0758** |
| 30 | 533 | 349.111 | 17 | Val-Cys-Lys | 1.17 | 0.0070 | **0.0236** |
| 31 | 1111 | 559.308 | 134 | N/A | 1.99 | 0.0104 | **0.0262** |
| 32 | 790 | 231.19 | 19 | N/A | 1.06 | 0.0148 | **0.0315** |
| 33 | 515 | 351.252 | 309 | N/A | 1.52 | 0.0148 | **0.0315** |
| 34 | 140 | 437.3 | 256 | N/A | 1.11 | 0.0148 | **0.0315** |
| 35 | 683 | 265.022 | 16 | N/A | 7.78 | 0.0263 | **0.0418** |
| 36 | 44 | 595.348 | 268 | N/A | 0.41 | 0.0207 | **0.0349** |
| 37 | 897 | 473.326 | 308 | Sumaresinolic acid | -0.79 | 0.0030 | **0.0218** |
| 38 | 984 | 458.371 | 308 | N/A | 2.27 | 0.0047 | **0.0218** |
| 39 | 960 | 176.105 | 18 | N/A | 2.44 | 0.0070 | **0.0236** |
| 40 | 501 | 471.309 | 254 | N/A | -0.61 | 0.0104 | **0.0262** |
| 41 | 1079 | 373.273 | 289 | Cholic Acid | -0.50 | 0.0047 | **0.0218** |
| 42 | 927 | 451.317 | 283 | N/A | 1.31 | 0.0499 | **0.0596** |
| 43 | 51 | 408.311 | 286 | Digoxigenin | 1.79 | 0.0281 | **0.0418** |
| 44 | 1126 | 399.145 | 16 | N/A | 4.29 | 0.0499 | **0.0596** |
| 45 | 1140 | 887.444 | 149 | N/A | 1.46 | 0.0148 | **0.0315** |
| 46 | 969 | 440.36 | 307 | N/A | 1.11 | 0.0047 | **0.0218** |
| 47 | 69 | 367.209 | 179 | N/A | 0.55 | 0.0499 | **0.0596** |
| 48 | 477 | 369.261 | 271 | N/A | 1.58 | 0.0207 | **0.0349** |
| 49 | 1004 | 592.319 | 231 | N/A | -0.44 | 0.0207 | **0.0349** |
| 50 | 729 | 490.324 | 239 | N/A | -0.75 | 0.0148 | **0.0315** |
| 51 | 715 | 614.292 | 194 | N/A | -0.49 | 0.0281 | **0.0418** |
| 52 | 685 | 592.321 | 195 | N/A | -0.44 | 0.0207 | **0.0349** |
| 53 | 484 | 591.317 | 267 | N/A | -0.45 | 0.0207 | **0.0349** |
| 54 | 734 | 260.196 | 20 | Leu-Lys | 1.13 | 0.0104 | **0.0262** |
| 55 | 338 | 591.317 | 232 | Urobilin | -0.43 | 0.0207 | **0.0349** |
| 56 | 1110 | 246.146 | 22 | Asn-Ile | 2.21 | 0.0104 | **0.0262** |
| 57 | 71 | 365.107 | 17 | N/A | 0.87 | 0.0047 | **0.0218** |
| 58 | 288 | 293.21 | 183 | N/A | 0.33 | 0.0379 | **0.0489** |
| 59 | 898 | 474.33 | 274 | N/A | -0.70 | 0.0047 | **0.0218** |
| 60 | 1119 | 732.384 | 165 | N/A | 17.55 | 0.0070 | **0.0236** |
| 61 | 365 | 202.071 | 18 | N/A | 0.60 | 0.0379 | **0.0489** |
| 62 | 204 | 485.327 | 238 | N/A | 4.84 | 0.0281 | **0.0418** |
| 63 | 266 | 455.356 | 262 | Corosolic Acid | 1.80 | 0.0047 | **0.0218** |
| 64 | 97 | 244.079 | 17 | Pro-Gln | 0.85 | 0.0070 | **0.0236** |
| 65 | 273 | 473.367 | 261 | N/A | 1.90 | 0.0070 | **0.0236** |
| 66 | 78 | 151.035 | 14 | N/A | 1.36 | 0.0281 | **0.0418** |
| 67 | 133 | 519.332 | 227 | N/A | 0.42 | 0.0379 | **0.0489** |
| 68 | 1124 | 495.787 | 126 | N/A | 112.84 | 0.0298 | **0.0434** |
| 69 | 594 | 246.133 | 25 | Asn-Ile | -0.68 | 0.0659 | **0.0760** |
| 70 | 965 | 656.409 | 250 | N/A | Inf | 0.0128 | **0.0313** |
| 71 | 899 | 260.185 | 262 | N/A | 1.66 | 0.2345 | 0.2369 |
| 72 | 656 | 851.397 | 290 | N/A | -0.99 | 0.0752 | **0.0847** |
| 73 | 197 | 485.326 | 293 | N/A | 1.84 | 0.0281 | **0.0418** |
| 74 | 1002 | 632.912 | 233 | N/A | -0.75 | 0.0301 | **0.0434** |
| 75 | 871 | 421.346 | 308 | N/A | 1.01 | 0.0104 | **0.0262** |
| 76 | 59 | 355.264 | 242 | Cholic Acid | 3.15 | 0.0148 | **0.0315** |
| 77 | 945 | 414.269 | 283 | N/A | -0.62 | 0.0207 | **0.0349** |
| 78 | 740 | 817.582 | 257 | Cholic Acid | -0.65 | 0.0379 | **0.0489** |
| 79 | 393 | 375.28 | 230 | N/A | -0.49 | 0.0379 | **0.0489** |
| 80 | 935 | 311.257 | 309 | 1,4-dihydroxyheptadec-16-en-2-yl acetate | 1.33 | 0.1049 | 0.1105 |
| 81 | 893 | 457.33 | 305 | N/A | -0.45 | 0.0499 | **0.0596** |
| 82 | 1011 | 303.133 | 182 | N/A | -0.32 | 0.0830 | **0.0924** |
| 83 | 19 | 434.189 | 26 | N/A | 1.47 | 0.0207 | **0.0349** |
| 84 | 233 | 491.246 | 21 | N/A | 1.61 | 0.0740 | **0.0843** |
| 85 | 645 | 489.32 | 214 | N/A | -0.63 | 0.0070 | **0.0236** |
| 86 | 1104 | 219.134 | 32 | Ser-Leu | 0.93 | 0.0104 | **0.0262** |
| 87 | 85 | 255.065 | 186 | Daidzin | 0.28 | 0.1049 | 0.1105 |
| 88 | 402 | 469.293 | 233 | N/A | -0.47 | 0.0379 | **0.0489** |
| 89 | 612 | 367.246 | 294 | N/A | 1.64 | 0.0207 | **0.0349** |
| 90 | 36 | 230.186 | 17 | Milnacipran | 0.89 | 0.0650 | **0.0758** |
| 91 | 363 | 577.29 | 132 | N/A | 1.91 | 0.1049 | 0.1105 |
| 92 | 244 | 506.265 | 151 | N/A | 1.25 | 0.1949 | 0.1989 |
| 93 | 188 | 257.081 | 186 | N/A | -0.42 | 0.1889 | 0.1949 |
| 94 | 220 | 217.068 | 17 | N/A | 0.77 | 0.0281 | **0.0418** |
| 95 | 206 | 466.291 | 196 | N/A | 1.37 | 0.1049 | 0.1105 |
| 96 | 1135 | 628.81 | 114 | N/A | 1.61 | 0.3717 | 0.3717 |
| 97 | 1093 | 473.369 | 262 | N/A | 1.39 | 0.0379 | **0.0489** |
| 98 | 332 | 568.224 | 223 | N/A | -0.28 | 0.1559 | 0.1626 |

**Day 4 Post-Sleep Disruption**

| VSURF Rank | Feature ID | M/Z | RT (sec) | Annotated Name | Fold Difference (Dis vs Con) | P Value | Adj. P Value |
| --- | --- | --- | --- | --- | --- | --- | --- |
| 1 | 238 | 621.293 | 189 | N/A | -0.22 | 0.0039 | **0.0622** |
| 2 | 697 | 343.175 | 181 | N/A | -0.58 | 0.0039 | **0.0622** |
| 3 | 894 | 437.304 | 280 | Hederagenin or N/A | -0.43 | 0.0005 | **0.0312** |
| 4 | 586 | 455.314 | 292 | Wilforlide A | -0.43 | 0.0011 | **0.0336** |
| 5 | 241 | 434.227 | 17 | N/A | 3.18 | 0.0101 | **0.0803** |
| 6 | 70 | 520.339 | 303 | Methyl 3-acetoxy-16-hydroxy-4,4,8,12,16-pentamethyl-15,17,19-trioxoandrost-11-ene-14-carboxylate | -0.47 | 0.0892 | 0.1903 |
| 7 | 62 | 412.236 | 196 | N/A | -0.20 | 0.1051 | 0.1922 |
| 8 | 524 | 293.21 | 220 | trans-EKODE-(E)-Ib | -0.26 | 0.0288 | 0.1317 |
| 9 | 739 | 391.245 | 305 | N/A | -0.78 | 0.0191 | 0.1109 |
| 10 | 579 | 456.318 | 306 | N/A | -0.24 | 0.0147 | **0.0940** |
| 11 | 980 | 990.591 | 118 | N/A | 99.00 | 0.0423 | 0.1457 |
| 12 | 522 | 594.401 | 222 | N/A | -0.31 | 0.0232 | 0.1144 |
| 13 | 638 | 391.215 | 271 | N/A | -0.07 | 0.1207 | 0.2019 |
| 14 | 661 | 287.244 | 16 | N/A | 5.39 | 0.0082 | **0.0803** |
| 15 | 1096 | 686.356 | 120 | N/A | 4.40 | 0.0752 | 0.1852 |
| 16 | 187 | 385.143 | 161 | N/A | -0.25 | 0.7913 | 0.8583 |
| 17 | 659 | 463.305 | 266 | N/A | -0.26 | 0.0892 | 0.1903 |
| 18 | 838 | 407.239 | 102 | N/A | -0.77 | 0.1028 | 0.1922 |
| 19 | 910 | 879.381 | 228 | N/A | -0.70 | 0.1655 | 0.2522 |
| 20 | 540 | 221.639 | 136 | N/A | 5.11 | 0.0535 | 0.1632 |
| 21 | 170 | 442.27 | 136 | N/A | 2.84 | 0.1230 | 0.2019 |
| 22 | 915 | 843.226 | 233 | N/A | -0.87 | 0.0968 | 0.1922 |
| 23 | 45 | 441.201 | 191 | N/A | -0.60 | 0.0753 | 0.1852 |
| 24 | 558 | 989.584 | 118 | N/A | 73.92 | 0.0518 | 0.1632 |
| 25 | 376 | 174.076 | 18 | N/A | 1.85 | 0.0355 | 0.1335 |
| 26 | 306 | 248.152 | 119 | N/A | 7.57 | 0.0113 | **0.0803** |
| 27 | 118 | 495.296 | 120 | N/A | 5.26 | 0.0892 | 0.1903 |
| 28 | 500 | 903.378 | 190 | N/A | -0.87 | 0.0355 | 0.1335 |
| 29 | 966 | 248.653 | 118 | N/A | 16.15 | 0.0695 | 0.1852 |
| 30 | 744 | 330.263 | 294 | N/A | -0.37 | 0.1051 | 0.1922 |
| 31 | 37 | 1191.503 | 184 | N/A | 0.08 | 0.4359 | 0.5166 |
| 32 | 754 | 455.311 | 306 | N/A | -0.22 | 0.0433 | 0.1457 |
| 33 | 557 | 883.532 | 135 | N/A | 26.43 | 0.0752 | 0.1852 |
| 34 | 912 | 281.104 | 189 | N/A | -0.84 | 0.0089 | **0.0803** |
| 35 | 716 | 470.34 | 289 | N/A | 0.15 | 0.4359 | 0.5166 |
| 36 | 928 | 364.27 | 191 | N/A | 0.39 | 0.4359 | 0.5166 |
| 37 | 1021 | 618.333 | 268 | N/A | -0.05 | 0.4813 | 0.5404 |
| 38 | 407 | 860.468 | 186 | N/A | -0.19 | 0.0632 | 0.1839 |
| 39 | 1047 | 279.1 | 31 | N/A | 13.66 | 0.1012 | 0.1922 |
| 40 | 284 | 333.203 | 308 | N/A | -0.54 | 0.0089 | **0.0803** |
| 41 | 275 | 457.329 | 283 | N/A | -0.09 | 0.8534 | 0.8810 |
| 42 | 532 | 295.226 | 225 | 13-Keto-9Z,11E-octadecadienoic acid | -0.13 | 0.0892 | 0.1903 |
| 43 | 889 | 471.306 | 239 | N/A | -0.28 | 0.1230 | 0.2019 |
| 44 | 509 | 692.341 | 182 | N/A | -0.29 | 0.1431 | 0.2290 |
| 45 | 251 | 215.139 | 22 | Pro-Val | -0.44 | 0.1230 | 0.2019 |
| 46 | 724 | 473.364 | 263 | N/A | -0.16 | 1.0000 | 1.0000 |
| 47 | 855 | 502.324 | 280 | N/A | -0.27 | 0.1655 | 0.2522 |
| 48 | 899 | 260.185 | 262 | N/A | 0.84 | 0.8534 | 0.8810 |
| 49 | 964 | 612.383 | 249 | N/A | Inf | 0.0350 | 0.1335 |
| 50 | 58 | 374.277 | 232 | N/A | -0.22 | 0.2799 | 0.3894 |
| 51 | 405 | 412.305 | 288 | N/A | -0.04 | 0.4813 | 0.5404 |
| 52 | 459 | 311.182 | 129 | N/A | 0.53 | 0.3930 | 0.5031 |
| 53 | 155 | 303.169 | 160 | N/A | -0.51 | 0.0232 | 0.1144 |
| 54 | 451 | 521.344 | 303 | N/A | -0.23 | 0.3930 | 0.5031 |
| 55 | 914 | 440.194 | 228 | N/A | -0.73 | 0.3068 | 0.4177 |
| 56 | 1103 | 378.239 | 160 | Val Leu Phe | 0.57 | 0.3527 | 0.4702 |
| 57 | 411 | 330.119 | 107 | N/A | 0.36 | 0.4359 | 0.5166 |
| 58 | 962 | 542.271 | 135 | N/A | 0.96 | 0.1903 | 0.2833 |
| 59 | 137 | 406.295 | 288 | N/A | -0.03 | 0.7394 | 0.8159 |
| 60 | 512 | 514.283 | 181 | N/A | 0.12 | 0.8534 | 0.8810 |
| 61 | 422 | 378.212 | 182 | N/A | 4.07 | 0.2083 | 0.3030 |
| 62 | 1079 | 373.273 | 289 | Cholic Acid | 0.07 | 0.4813 | 0.5404 |
| 63 | 116 | 379.295 | 236 | N/A | -0.17 | 0.2475 | 0.3519 |
| 64 | 1026 | 862.64 | 256 | N/A | 5.39 | 0.8815 | 0.8955 |

**Table S3. VSURF Suprathreshold Metabolites:** Variable Selection Using Random Forests (VSURF) was performed at day 2 post-sleep disruption (R2) and day 4 post-sleep disruption (R4) to identify top drivers of separation between sleep-disrupted and control groups. Metabolites that were above the VSURF threshold variable importance (‘suprathreshold’) from each timepoint are listed above, along with the feature’s ID, m/z ratio, retention time, annotated name (if any), fold difference in the sleep-disrupted group compared to the control group, unadjusted Wilcoxon Rank-Sum *p* value, and Benjamini Hochberg (FDR)-adjusted *p* value. Bold indicates adjusted *p* values that are below the FDR of 0.1. Fold difference: (Sleep Disruption-Control)/Control. Abbreviations: Dis, sleep disruption; Con, control; MZ, mass to charge ratio; RT, retention time; N/A, not annotated; Inf, infinity. *n* = 8-10/group.
