# Supplementary material for: Repeated sleep disruption in mice leads to persistent shifts in the fecal microbiome and metabolome
Source: PLoS One. 2020 Feb 20;15(2):e0229001. doi: 10.1371/journal.pone.0229001 (PMC7032712; doi:10.1371/journal.pone.0229001)
Supplement: Attachment — Submitted filename: Response to Reviewers.docx [file pone.0229001.s011.docx]

Response to Reviewers

Manuscript: PONE-D-19-35357
Repeated sleep disruption in mice leads to persistent shifts in the fecal microbiome and metabolome

Please see below for a point-by-point response to the reviewer’s comments.

Comments from Reviewer #1:

- **“Throughout the manuscript: Stick to microbiome rather than microbiota”**
  - We have updated the terminology used in the manuscript accordingly.
- **“L595, L637: Provide the experimental model for these studies”**
  - We agree with the reviewer that it is important to describe the type of sleep disruption protocols used in the studies we cite at these locations in the paper. Therefore, we have amended and expanded the text to do so:

Line 595 - Pre-revision:

Post-revision:

Line 637 – Pre-revision:

Post-revision:
